# An ultra-rapid mounting method for *C. elegans* embryos that enables long-term, multi-sample live imaging

**DOI:** 10.17912/micropub.biology.002361

**Published:** 2026-09-17

**Authors:** Yuzuha Komachiya, Akane Matsumura, Natsu Sato, Riho Kato, Hayao Ohno

**Affiliations:** 1 Division of Material and Biological Sciences, Graduate School of Science, Japan Women’s University, Tokyo, Japan; 2 Department of Chemical and Biological Sciences, Faculty of Science, Japan Women’s University, Tokyo, Japan

## Abstract

Live imaging of *Caenorhabditis elegans* embryos often requires labor-intensive dissection or mounting procedures. Here we present a versatile method in which nematode growth medium (NGM) agar chunks containing laid embryos are excised and inverted directly onto coverslips or chamber slides. Preparation requires less than one minute, enabling immediate fluorescence and transmitted-light microscopy on both inverted and upright systems. Moisture loss is minimized by using a protective cap or chamber lid, permitting uninterrupted 24-hour time-lapse imaging. Combining multi-well slides with a motorized stage allows simultaneous multi-sample tracking. This rapid approach streamlines live-imaging genetic screens and developmental analyses.

**Figure 1. Simple, rapid mounting of C. elegans embryos by placing an NGM agar chunk on a coverslip or into a multi-well chamber slide:** ( **A** ) Sequence of photographs: excision of an NGM agar chunk with embryos laid on it using flat-tip tweezers (left), inversion of the agar chunk (center), and placement of the inverted chunk onto a large coverslip (right). ( **B** ) Photograph of the slide prepared as in (A) being observed through the coverslip on an inverted compound microscope. ( **C** ) Schematic of embryo observation using an inverted microscope for specimens mounted as in (A). ( **D** and **E** ) Representative embryo at the 1.5-fold stage from the strain CAT140 ( *stIs10453* *ujIs113* ) imaged using the method in (A–C). (D) Bright-field image and (E) fluorescence image showing ELT-2::GFP expressed in endodermal cells (green) and mCherry::Histone expressed in all cells (magenta). Scale bar, 10 µm. ( **F** ) Photograph showing a cut-off 5-mL centrifuge-tube cap placed over the agar chunk on the coverslip prepared as in (A) to maintain a humid environment during long-term imaging. ( **G** ) Example time-lapse of N2 embryos mounted using the cap-covered arrangement in (F) and followed for 24 h; preventing drying enables continuous imaging from early embryogenesis through hatching and progression to the L2 larval stage. Scale bar, 100 µm. ( **H** ) Photograph of an inverted slide prepared as in (A), placed onto the lid of a 3.5-cm plastic Petri dish. ( **I** ) Photograph of the slide prepared as in (H) being observed through the coverslip with an upright compound microscope. ( **J** ) Schematic of embryo observation using an upright microscope for specimens mounted as in (H). ( **K** ) Photograph showing the attachment of an NGM agar chunk to a coverslip using transparent tape. ( **L** ) Photograph of an 8-well chamber slide in which inverted NGM agar chunks were placed in each well as in (A), imaged with an inverted microscope. When combined with a motorized stage that can store stage coordinates, this configuration enables simultaneous time-lapse imaging of eight samples. ( **M** ) Schematic of embryo observation using an inverted microscope for specimens mounted as in (L).

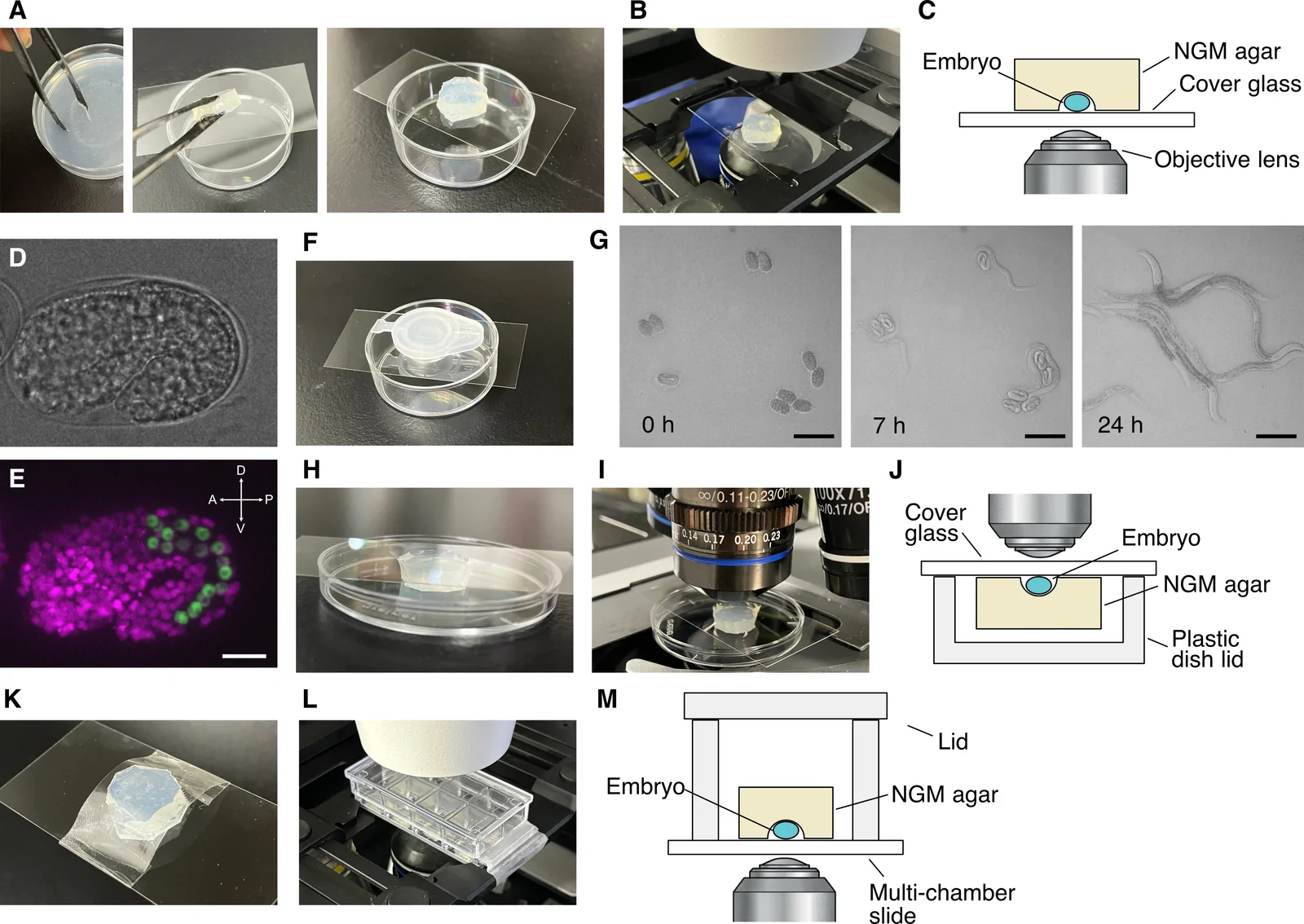



## Description

The nematode *Caenorhabditis elegans* is an ideal model for studying embryogenesis because both the eggshell and the embryo are transparent, the complete cell lineage of embryonic development is known (Sulston et al., 1983), and a wide range of fluorescent transgenic lines are readily available and can be generated with relative ease. Nevertheless, the small size of *C. elegans* embryos (approximately 50 µm along their long axis) makes them harder to isolate from culture plates and to examine under a compound microscope than post-hatch larvae or adults. Washing plates with buffer does not reliably retrieve embryos because most remain attached to the agar surface. In addition, the embryos' near-spherical geometry necessitates controlled, mild compression along the optical axis to position deep cells within the working distance of the objective. Common mounting approaches include dissecting gravid adults to release eggs onto an agar pad (Walston and Hardin, 2010) or isolating embryos and sandwiching them between two coverslips with microplastic beads before sealing (Bao and Murray, 2011). These techniques require substantial practice to perform efficiently and take considerable time even for skilled practitioners. Consequently, performing large-scale genetic screens that depend on compound-microscope observation of embryos—such as ethyl methanesulfonate (EMS) mutagenesis screens or RNAi screens—or simultaneously imaging many different strains is technically challenging.

Here we describe an exceptionally rapid mounting method for observing *C. elegans* embryos with a compound microscope. Sterile flat-tip tweezers are used to cut a piece of NGM agar containing laid embryos from the culture plate; this agar piece is flipped onto a large coverslip and gently compressed ( Fig. 1A ), and embryos are then observed from beneath using an inverted microscope ( Fig. 1B, C). A single coverslip can accommodate several agar pieces simultaneously. The simplicity of the protocol allows imaging to begin in less than one minute, making it particularly useful for quick pilot checks and for high-throughput genetic screens focused on embryonic phenotypes. In transmitted-light imaging, the agar piece lies in the path of transmitted light entering from the side opposite the objective ( Fig. 1C ), which might be expected to interfere with illumination; nonetheless, because the NGM agar is translucent, the overall embryo morphology remains readily visible ( Fig. 1D ). Fluorescence imaging, in which excitation light is delivered through the objective, can be performed in the same manner as on a conventional glass slide ( Fig. 1E ); for example, increases in embryonic intestinal cell numbers arising from maternal exposure to harmful gut microbes (Ohno and Bao, 2022)—a reported epigenetic adaptation—can readily be detected.

With the approach shown in Fig. 1A, the agar tends to dry out after 3–6 hours, which can render continued observation impossible. Placing a cut-off 5-mL centrifuge tube cap (for example, Eppendorf, Cat. No. 0030119460) over the agar piece ( Fig. 1F ) prevents desiccation and extends the imaging duration. Using this cap-covered arrangement, the agar maintains its shape for at least 24 hours, allowing continuous time-lapse imaging from early embryogenesis through hatching and even progression to the L2 larval stage ( Fig. 1G ). To minimize interference from motile post-hatch worms and residual bacteria during prolonged imaging, the surface of the NGM culture plate can be rinsed with buffer prior to excising agar pieces. This step lowers the number of post-hatch worms and food bacteria without dislodging embryos from the agar.

When only an upright microscope is available, specimens may be prepared by lightly pressing the NGM agar piece onto a coverslip and then flipping the coverslip for observation ( Fig. 1H –J); the agar does not fall away from the coverslip, but we recommend placing the slide on the lid of a 3.5-cm plastic Petri dish as a precaution ( Fig. 1H –J). If the agar shifts downward under gravity and the embryos are not sufficiently immobilized, apply a small piece of transparent tape to affix the agar to the coverslip before inverting ( Fig. 1K ).

Finally, when simultaneous imaging of many different samples on a single microscope is required, individual inverted NGM agar chunks can be placed into the wells of a multi-well chamber slide (for example, Matsunami, Cat. No. SCC-008) ( Fig. 1L, M). Using a chamber slide with a lid prevents the agar pieces from desiccating, allowing long-term time-lapse imaging equivalent to the cap-covered arrangement shown in Fig. 1F . On an inverted microscope equipped with a motorized stage, an 8-well chamber slide potentially enables the parallel tracking of development for hundreds of embryos across eight strains. This approach streamlines large-scale assays, such as measuring embryonic hatching times (e.g., Liu et al., 2012) across many mutants.

The limitations of these methods include: (1) At least in wild-type strains, it is difficult to find embryos immediately after fertilization by inspecting the surface of a culture plate directly. While embryos at the 4-cell stage are occasionally found on a plate, observing embryos at earlier stages generally requires either the use of egg-laying constitutive (Egl-c) mutants or manual dissection of gravid adults to release embryos. (2) Light scattering by the agar chunk and the presence of food microbes on the medium can reduce image contrast and resolution, particularly for transmitted-light microscopy; therefore, this mounting strategy is unlikely to be appropriate for experiments that rely solely on differential interference contrast (DIC) imaging to identify cells, as was common before the advent of fluorescent proteins. (3) Retrieving a specific embryo after imaging can be challenging. Although possible in most cases, peeling agar pieces off the coverslip can lead to loss or misplacement of embryos, especially when many are mounted.

## Methods

Worms were maintained using standard methods (Brenner, 1974) on *E. coli* HB101 . For imaging, a region of NGM agar containing embryos was cut out with flat-tip tweezers into pieces approximately 5–20 mm on a side and inverted onto either a 24 × 50 mm coverslip (Matsunami, Cat. No. C024501), a 25 × 36 mm coverslip (Matsunami, Cat. No. C025361), or an 8-well glass-bottom chamber (Matsunami, Cat. No. SCC-008). Imaging was carried out with 20× (Evident, UPLXAPO20X, dry, NA = 0.95) or 60× (Evident, UPLSAPO60XW, water-immersion, NA = 1.2) objectives. The imaging platform was a motorized inverted fluorescence microscope consisting of an Axio Observer 7 (Zeiss), an ORCA-Fusion BT camera (Hamamatsu Photonics), a Colibri 7 light source (Zeiss), an Apotome 3 structured-illumination module (Zeiss), and a Scanning Stage 130×100 STEP (Zeiss). A 5-mL centrifuge tube cap from Eppendorf (Cat. No. 0030119460) was used in the procedure.

## Reagents

Strains used in this study: 

**Table:** 

N2	*Caenorhabditis elegans* wild isolate.
CAT140	*stIs10453* [ *elt-2::TGF(7E1)::GFP::TY1::3xFLAG inserted into fosmid WRM0617dE06 as C-terminal protein fusion* ] *ujIs113* [ *pie-1prom::mCherry::H2B* , *nhr-2prom::mCherry::HIS-24::let-858UTR* , *unc-119 (+)* ] *II* .
